# Tunable acoustic scattering in composite duct structures with elastic membrane discontinuities

**DOI:** 10.1371/journal.pone.0332960

**Published:** 2025-10-06

**Authors:** Hani Alahmadi, Aqsa Yaseen, Salman Saud Alsaeed, Muhammad Afzal, Naif Alkuhayli

**Affiliations:** 1 Department of Mathematics, College of Science, Jouf University, Sakaka, Saudi Arabia; 2 Department of Mathematics, COMSATS University Islamabad, Tarlai Kalan, Islamabad, Pakistan; 3 Department of Mathematics and Natural Sciences, Center for Applied Mathematics and Bioinformatics, Gulf University for Science & Technology, Hawally, Kuwait; Beni-Suef University, EGYPT

## Abstract

This study investigates acoustic wave scattering in a composite duct structure incorporating a rigid annular shell connected to a flexible cylindrical shell via an embedded elastic membrane disc. The presence of the membrane introduces a vibratory discontinuity that significantly influences acoustic propagation and scattering characteristics. We develop a semi-analytical framework combining the Mode Matching (MM) technique for rigid-fluid interfaces with a Galerkin-based approach to model the elastic membrane vibrations. The formulation enforces continuity of pressure and axial velocity at the fluid-structure interfaces through truncated modal expansions, with convergence rigorously verified. By varying membrane edge constraints (spring-like, clamped, free) and geometric parameters, we identify distinct scattering regimes and demonstrate how structural and boundary variations can enhance or suppress acoustic reflection. Under spring-like conditions, a transmission loss of 13.5 dB is achieved, with subsequent variations governed by changes in scattering powers due to different parametric settings and edge conditions. The findings provide insights for the design of advanced tunable acoustic devices, including silencers and metamaterial waveguides, where controlling wave scattering is critical.

## 1 Introduction

Acoustic wave propagation and scattering in duct-like structures containing structural discontinuities remain critical in the design and optimization of noise control systems, acoustic liners, and waveguides [1–3]. Traditional studies have extensively focused on rigid discontinuities and impedance discontinuities in axisymmetric configurations [4–8], yet the presence of flexible or vibrating elements introduces complex fluid-structure interactions that require refined mathematical treatment. A substantial body of literature has addressed structural-acoustic coupling in cylindrical shells and ducts with vibrating walls or flexible linings. Early work by Fuller [9], and later developments by Wang and Lai [10], explored sound radiation from elastic shells, underscoring the role of geometry and wall compliance. Similar insights have been reported in the context of fluid-loaded, axisymmetric pipes and shells with varying boundary conditions [11,12]. Furthermore, the vibrational behavior of elastic shells and membrane components under varying boundary constraints has been rigorously studied through classical shell theories [13,14], which form the theoretical backbone for complex vibroacoustic modeling.

Despite this progress, there remains a lack of comprehensive studies that analyze the impact of embedded elastic elements such as circular membrane discs, within duct systems that also include transitions between rigid and flexible domains. The conventional solutions such as rigid wall linings, porous absorbers, and Helmholtz resonators are often effective only over narrow frequency ranges and may require large installation volumes to target low frequency noise. Moreover, these treatments typically assume rigid or uniformly compliant boundaries, overlooking the complex fluid structure coupling that arises when flexible or partially constrained elements are embedded within otherwise rigid systems. More recent investigations have started addressing this gap by integrating hybrid analytical-numerical methods like Mode Matching (MM) and Galerkin formulations to evaluate scattering in discontinuous waveguides involving both geometrical and material changes [15–19]. Lawrie and Abraham [20] studied a membrane-bounded duct using the mode-matching technique combined with generalized orthogonality relations. Warren et al. [21] analyzed a rigid step discontinuity at the interface employing the mode-matching approach and validated their results by comparison with the Wiener–Hopf method for planar geometries. The mode-matching method depends on the properties of eigenfunctions, whose analytical characteristics for flexible bounded ducts were rigorously established in [22].

The present work extends this line of inquiry by examining acoustic scattering from a composite structure comprising a membrane disc junctions that connect a rigid annular shell to a flexible cylindrical shell. The embedded membrane disc introduces an elastic, vibratory discontinuity whose acoustic influence depends strongly on edge constraints, material parameters, and radial extent. The earlier studies like [15] have largely focused on rectangular configurations with flexible wall segments or localized panels, often with simplified junction treatments. In contrast, the present study addresses a three-dimensional cylindrical duct system with bifurcated inlet and outlet branches and an expansion chamber that contains a flexible shell spanning its length. The shell is modeled using the Donnell–Mushtari equations [13,23] to capture both longitudinal and transverse displacements, while the interfaces between the chamber and adjoining waveguides are occupied by thin elastic membrane discs that replace rigid discontinuities. These membranes are modeled separately, allowing for fixed, free, and impedance-type edge conditions, and are coupled to the acoustic field through pressure and velocity continuity. The analytical framework combines mode matching for the acoustic field with a Galerkin projection for the structural dynamics, enabling accurate treatment of the coupled interaction between the cylindrical waveguide, the flexible shell, and the membranes. Note that eigenfunctions in the region bounded by flexible shell are non-orthogonal [24–27] and satisfy the generalized form of eigenfunction properties and ensure the point wise convergence of the solution [22].

The motivation for this study arises from the need for tunable and efficient acoustic control devices in applications such as silencers, noise abatement systems, and acoustic metamaterial waveguides. Conventional duct designs with rigid boundaries often provide limited adaptability, whereas incorporating elastic elements allows the scattering and transmission characteristics to be tailored to specific frequency ranges. In the present configuration, a cylindrical composite duct with bifurcated branches contains a flexible shell in the expansion chamber and elastic membrane discs at the interfaces. We develop a semi-analytical formulation that combines the MM method for rigid transitions with a Galerkin-based approach for the elastic membrane. Special attention is paid to satisfying the continuity of pressure and axial velocity at the membrane–fluid interfaces using a truncated modal expansion strategy, validated through convergence studies. By systematically varying the edge constraints (e.g., spring-like, clamped, and free edges) and geometric parameters (e.g., membrane radius), the study uncovers distinct scattering regimes and identifies configurations conducive to enhanced or suppressed wave reflection. The findings have practical implications for the design of tunable acoustic devices such as silencers, metamaterial-based waveguides, and advanced liner technologies.

The structure of this article is organized to systematically guide the reader through the development and analysis of the proposed acoustic scattering model. Sect 2 introduces the mathematical formulation of the physical problem, detailing the governing equations and boundary conditions. In Sect 3, the Mode Matching technique is presented as the primary method to address wave propagation and scattering at the rigid-fluid interfaces. Sect 4 elaborates on the Galerkin formulation used to model the vibratory behavior of the elastic membrane disc embedded within the duct system. Numerical results illustrating the effects of varying edge constraints and geometric parameters are discussed in Sect 5, highlighting key scattering phenomena and validation of the computational approach. Finally, Sect 6 summarizes the main conclusions and discusses potential applications and future research directions.

## 2 Formulation of the boundary value problem

**Fig 1:**
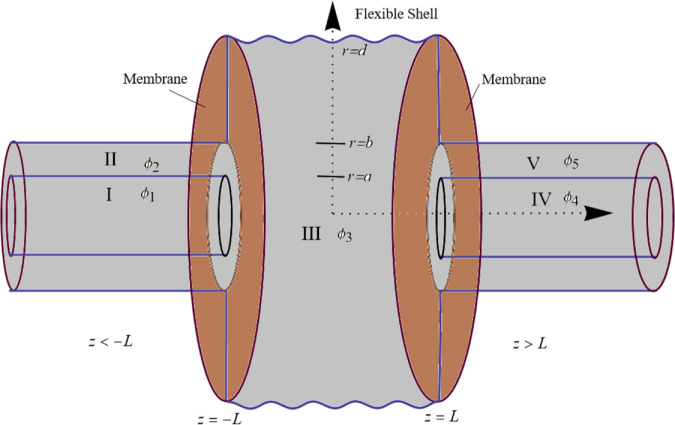
Waveguide geometry.

We investigate fluid–structure wave propagation in a cylindrical waveguide filled with a compressible fluid of density *ρ* and sound speed *c*. The waveguide consists of segments with different boundary materials. The inlet and outlet sections are rigid, incapable of supporting structural vibrations, and include bifurcations. These rigid, coaxial cylindrical ducts have dimensional radii $\bar{a}$ and $\bar{b}$ at the inlet and outlet, respectively, with $\bar{b}>\bar{a}$. They are connected to a central flexible shell of radius $\bar{d}$ via membrane discs located at the interfaces. Throughout the formulation, dimensional quantities are denoted by an overbar. The physical configuration comprises five regions, labeled I through V, as illustrated in Fig 1. Incident acoustic waves enter through ducts I and II (the inlets), propagate through the flexible shell region (duct III), and exit through ducts IV and V (the outlets). The dimensional fluid potential $\bar{\Phi }$ within the waveguide satisfies the Helmholtz equation, along with boundary and interface conditions dictated by the flexible shell and membrane discs. Harmonic time dependence is assumed, such that $\bar{\Phi }=\bar{\phi }\;e^{-i\omega \bar{t}}$, where ω=ck is the angular frequency and k=ω/c is the wavenumber. In cylindrical coordinates $\left(\bar{r},\bar{\theta },\bar{z}\right)$, the governing equations are:

- **Helmholtz equation in all fluid regions:**
  $$\left\{\frac{\partial^{2}}{\partial {\bar{r}}^{2}}+\frac{1}{\bar{r}}\frac{\partial }{\partial \bar{r}}+\frac{1}{{\bar{r}}^{2}}\frac{\partial^{2}}{\partial {\bar{\theta }}^{2}}+\frac{\partial^{2}}{\partial {\bar{z}}^{2}}+k^{2}\right\}\bar{\phi }=0,$$ (1)
- **Rigid-wall boundary conditions:**
  $$\frac{\partial \bar{\phi }}{\partial \bar{r}}=0,\;\bar{r}=\bar{a},\bar{b},\;|\bar{z}|>\bar{L}.$$ (2)
- **Flexible shell dynamics** are modeled using the Donnell–Mushtari shell theory, which is applicable to structures with small thickness-to-radius ratios and moderate frequency ranges, where bending and in-plane stretching are the dominant deformation modes. At $\bar{r}=\bar{d}$ and $|\bar{z}|<\bar{L}$, the equations of motion are:
  $$\frac{\partial^{2}\bar{u}}{\partial {\bar{z}}^{2}}+\frac{1-\nu }{2{\bar{d}}^{2}}\frac{\partial^{2}\bar{u}}{\partial {\bar{\theta }}^{2}}+\frac{1+\nu }{2\bar{d}}\frac{\partial^{2}\bar{v}}{\partial \bar{z}\partial \bar{\theta }}+\frac{\nu }{\bar{d}}\frac{\partial \bar{w}}{\partial \bar{z}}+\frac{\omega^{2}\bar{u}}{c_{s}^{2}}=0,$$ (3)
  $$\frac{1+\nu }{2\bar{d}}\frac{\partial^{2}\bar{u}}{\partial \bar{z}\partial \bar{\theta }}+\frac{1-\nu }{2}\frac{\partial^{2}\bar{v}}{\partial {\bar{z}}^{2}}+\frac{1}{{\bar{d}}^{2}}\frac{\partial^{2}\bar{v}}{\partial {\bar{\theta }}^{2}}+\frac{1}{{\bar{d}}^{2}}\frac{\partial \bar{w}}{\partial \bar{\theta }}+\frac{\omega^{2}\bar{v}}{c_{s}^{2}}=0,$$ (4)
  $$\frac{\nu }{\bar{d}}\frac{\partial \bar{u}}{\partial \bar{z}}+\frac{1}{{\bar{d}}^{2}}\frac{\partial \bar{v}}{\partial \bar{\theta }}+\frac{\bar{w}}{{\bar{d}}^{2}}+\frac{{\bar{h}}^{2}}{12}\frac{\partial^{4}\bar{w}}{\partial {\bar{z}}^{4}}+\frac{2{\bar{h}}^{2}}{12{\bar{d}}^{2}}\frac{\partial^{4}\bar{w}}{\partial {\bar{z}}^{2}\partial {\bar{\theta }}^{2}}+\frac{{\bar{h}}^{2}}{12{\bar{d}}^{4}}\frac{\partial^{4}\bar{w}}{\partial {\bar{\theta }}^{4}}\;\;-\frac{\omega^{2}\bar{w}}{c_{s}^{2}}-\frac{\bar{p}(\bar{d},\bar{z})}{c_{s}^{2}\rho_{s}\bar{h}}=0,$$ (5)
  where $\bar{u}$, $\bar{v}$, and $\bar{w}$ represent the longitudinal, circumferential, and radial displacements of the shell, and $\bar{p}$ is the acoustic pressure. The wave speed in the shell is $c_{s}=\sqrt{E/\left((1-\nu^{2})\rho_{s}\right)}$, where $\rho_{s}$ is the shell density, ν is Poisson’s ratio, and *E* is Young’s modulus. The flexible shell is clamped at its connections to the rigid ducts at $\bar{z}=\pm \bar{L}$ and $\bar{r}=\bar{d}$, satisfying:
  $$\bar{u}=\bar{v}=\frac{\partial \bar{\phi }}{\partial \bar{r}}=\frac{\partial^{2}\bar{\phi }}{\partial \bar{r}\partial \bar{z}}=0.$$ (6)
- **Membrane disc dynamics**, in the annular region $\bar{b}<\bar{r}<\bar{d}$ at $\bar{z}=\pm \bar{L}$: The radial displacement $\bar{q}$ of the membrane satisfies:
  $$\frac{\partial^{2}\bar{q}}{\partial {\bar{r}}^{2}}+\frac{\omega^{2}}{c_{m}^{2}}\bar{q}=\frac{1}{T}{\left[\bar{p}\right]}_{-}^{+},$$ (7)
  where $c_{m}=\sqrt{T/\rho_{m}}$ is the wave speed on the membrane, *T* is the membrane tension, and $\rho_{m}$ is the mass per unit area. The term ${\left[\bar{p}\right]}_{-}^{+}={\bar{p}}^{+}$ − ${\bar{p}}^{-}$ represents the jump in acoustic pressure across the membrane. At the membrane edges ($\bar{r}=\bar{b},\bar{d}$), the displacement satisfies spring-type boundary conditions:
  $$\bar{\sigma }\bar{q}+\frac{\partial \bar{q}}{\partial \bar{r}}=0,\;\bar{z}=\pm \bar{L},\;\bar{r}=\bar{b},\bar{d},$$ (8)
  where $\bar{\sigma }$ is the dimensional coupling constant.

To facilitate the analysis, we nondimensionalize the problem using the transformations $r=k\bar{r}$, $z=k\bar{z}$, and $\phi =k^{2}\bar{\phi }/\omega$. The resulting dimensionless formulation is then solved using the mode-matching method and Galerkin approach, as detailed in the following sections.

## 3 Mode-matching solution

In the mode-matching approach, we first derive modal solutions using the method of separation of variables and then impose interface matching conditions. This transforms the differential boundary value problem into a linear algebraic system, which is subsequently truncated and solved numerically. The complete mode-matching solution is presented in the following subsections.

### 3.1 Eigenfunction expansions in duct regions

To solve the dimensionless boundary value problem, the velocity potential in each duct region (I to V) is expressed in terms of eigenfunction expansions as follows:

- **Regions I and IV (|z|>L, 0 < *r* < *a*):** For axisymmetric modes, the velocity potentials are given by
  $$\phi_{1}(r,z)=e^{i(z+L)}+\sum_{n=0}^{\infty }A_{n}R_{1}(\tau_{n},r)e^{-i\eta_{n}(z+L)},$$ (9)
  $$\phi_{4}(r,z)=\sum_{n=0}^{\infty }E_{n}R_{1}(\tau_{n},r)e^{i\eta_{n}(z-L)},$$ (10)
  where $\eta_{n}=\sqrt{1-\tau_{n}^{2}}$ is the axial wavenumber corresponding to the radial eigenvalue $\tau_{n}$, which satisfies the condition $\tau J_{1}(\tau a)=0$. Here, $J_{1}(\cdot )$ is the Bessel function of the first kind and order one, and the associated eigenfunctions are
  $$R_{1}(\tau_{n},r)=J_{0}(\tau_{n}r),\;n=0,1,2,\ldots ,$$
  which satisfy the orthogonality relation
  $$\int_{0}^{a}R_{1}(\tau_{n},r)R_{1}(\tau_{m},r)r\;dr=\delta_{mn}M_{n},$$ (11)
  with normalization constant $M_{n}=\int_{0}^{a}R_{1}^{2}(\tau_{n},r)r\;dr$.
- **Regions II and V (|z|>L, *a* < *r* < *b*):** For axisymmetric modes, the velocity potentials are given by
  $$\phi_{2}(r,z)=e^{i(z+L)}+\sum_{n=0}^{\infty }B_{n}R_{2}(\gamma_{n},r)e^{-is_{n}(z+L)},$$ (12)
  $$\phi_{5}(r,z)=\sum_{n=0}^{\infty }F_{n}R_{2}(\gamma_{n},r)e^{is_{n}(z-L)},$$ (13)
  where $s_{n}=\sqrt{1-\gamma_{n}^{2}}$ is the axial wavenumber and $\gamma_{n}$ are the eigenvalues determined from the condition
  $$J_{1}(\gamma b)N_{1}(\gamma a)-J_{1}(\gamma a)N_{1}(\gamma b)=0.$$
  Here, $J_{1}(\cdot )$ and $N_{1}(\cdot )$ are Bessel functions of the first and second kind, respectively, both of order one. The associated eigenfunctions are
  $$R_{2}(\gamma_{n},r)=J_{0}(\gamma_{n}r)N_{1}(\gamma_{n}a)-J_{1}(\gamma_{n}a)N_{0}(\gamma_{n}r),$$
  where $N_{0}(\cdot )$ is the Bessel function of the second kind of order zero. These eigenfunctions satisfy the orthogonality relation
  $$\int_{a}^{b}R_{2}(\gamma_{n},r)R_{2}(\gamma_{m},r)r\;dr=\delta_{mn}O_{n},$$ (14)
  with $O_{n}=\int_{a}^{b}R_{2}^{2}(\gamma_{n},r)r\;dr$.
- **Region III (|z|<L, 0 < *r* < *d*):** For axisymmetric modes, the velocity potential and radial shell displacements are given by
  $$\phi_{3}(r,z)=\sum_{n=0}^{\infty }\left(C_{n}e^{i\lambda_{n}z}+D_{n}e^{-i\lambda_{n}z}\right)R_{3}(\xi_{n},r),$$ (15)
  $$u(d,z)=-\sum_{n=0}^{\infty }\frac{i\nu \lambda_{n}R_{3}^{\prime }(\xi_{n},d)}{(\lambda_{n}^{2}-\beta^{2})d}\left(C_{n}e^{i\lambda_{n}z}-D_{n}e^{-i\lambda_{n}z}\right),$$ (16)
  $$w(d,z)=\sum_{n=0}^{\infty }R_{3}^{\prime }(\xi_{n},d)\left(C_{n}e^{i\lambda_{n}z}+D_{n}e^{-i\lambda_{n}z}\right),$$ (17)
  where $\lambda_{n}=\sqrt{1-\xi_{n}^{2}}$ and the eigenvalues $\xi_{n}$ are roots of the dispersion relation
  $$-\Gamma \nu^{2}(1-\xi^{2})\xi J_{1}(\xi d)+\left[(1-\xi^{2})-\beta^{2}\right]\left[(1-\xi^{2}{)}^{2}-\mu^{4}\right]\xi J_{1}(\xi d)\;\;+\left[(1-\xi^{2})-\beta^{2}\right]\alpha J_{0}(\xi d)=0,$$ (18)
  with parameters $\mu^{4}=\Gamma (d^{2}\beta^{2}-1)$ and $\alpha =\frac{12\beta^{2}\rho }{\rho_{s}h^{3}k^{3}}$. The eigenfunctions
  $$R_{3}(\xi_{n},r)=J_{0}(\xi_{n}r),\;n=0,1,2,\ldots ,$$
  satisfy the generalized orthogonality condition [24]:
  $$\frac{\alpha }{d}\int_{0}^{d}R_{3}(\xi_{n},r)R_{3}(\xi_{m},r)r\;dr=\delta_{mn}X_{n}\;\;-\left[\frac{\Gamma \nu^{2}\beta^{2}}{\left(\lambda_{n}^{2}-\beta^{2})(\lambda_{m}^{2}-\beta^{2}\right)}+2-\xi_{n}^{2}-\xi_{m}^{2}\right]\;\;\times R_{3}^{\prime }(\xi_{n},d)R_{3}^{\prime }(\xi_{m},d),$$ (19)
  where
  $$X_{n}=\frac{\alpha d}{2}\left[J_{0}^{2}(\xi_{n}d)+J_{1}^{2}(\xi_{n}d)\right]+\left(\frac{\Gamma \nu^{2}\beta^{2}}{(\lambda_{n}^{2}-\beta^{2}{)}^{2}}+2\lambda_{n}^{2}\right){\left[R_{3}^{\prime }(\xi_{n},d)\right]}^{2}.$$ (20)

The term term generalized is used because of the involvement of the additional term on the right hand side of (19) and this makes the eigenfunctions linearly dependent, for more detail see [22]. Note that the modal expansions contain unknown coefficients *A*_*n*_, *B*_*n*_, …, *F*_*n*_, which are determined by enforcing continuity of acoustic pressure and normal velocity across the interfaces between regions. The next subsection presents the procedure for applying the matching conditions and solving for these modal amplitudes.

### 3.2 Modal matching at the interfaces

To determine the unknown modal coefficients, continuity conditions are imposed at the interfaces z=±L. These conditions ensure the continuity of acoustic pressure (i.e., velocity potential) across the boundaries between adjacent domains. Accordingly, we require that

$$\phi_{1}(r,-L)=\phi_{3}(r,-L),\;\;\;0\leq r\leq a,$$ (21)

$$\phi_{4}(r,L)=\phi_{3}(r,L),\;\;\;0\leq r\leq a,$$ (22)

$$\phi_{2}(r,-L)=\phi_{3}(r,-L),\;\;\;a\leq r\leq b,$$ (23)

$$\phi_{5}(r,L)=\phi_{3}(r,L),\;\;\;a\leq r\leq b.$$ (24)

To extract the modal coefficients, we multiply Eqs (21) and (22) by the weighting function $rR_{1}(\tau_{m},r)$ and integrate over the inner radial domain [0,*a*]. Similarly, Eqs (23) and (24) are multiplied by $rR_{2}(\gamma_{m},r)$ and integrated over the annular domain [*a*,*b*]. This yields:

$$\int_{0}^{a}\phi_{1}(r,-L)R_{1}(\tau_{m},r)\;r\;dr=\int_{0}^{a}\phi_{3}(r,-L)R_{1}(\tau_{m},r)\;r\;dr,$$ (25)

$$\int_{0}^{a}\phi_{4}(r,L)R_{1}(\tau_{m},r)\;r\;dr=\int_{0}^{a}\phi_{3}(r,L)R_{1}(\tau_{m},r)\;r\;dr,$$ (26)

$$\int_{a}^{b}\phi_{2}(r,-L)R_{2}(\gamma_{m},r)\;r\;dr=\int_{a}^{b}\phi_{3}(r,-L)R_{2}(\gamma_{m},r)\;r\;dr,$$ (27)

$$\int_{a}^{b}\phi_{5}(r,L)R_{2}(\gamma_{m},r)\;r\;dr=\int_{a}^{b}\phi_{3}(r,L)R_{2}(\gamma_{m},r)\;r\;dr.$$ (28)

Substituting the modal expansions defined in Eqs (9)–(13) into (25)–(28), and utilizing the orthogonality relations (11) and (14), we obtain the following expressions for the modal amplitudes:

$$A_{m}=-\delta_{m0}+\frac{1}{M_{m}}\sum_{n=0}^{\infty }\left(C_{n}e^{-i\lambda_{n}L}+D_{n}e^{i\lambda_{n}L}\right)P_{mn},$$ (29)

$$E_{m}=\frac{1}{M_{m}}\sum_{n=0}^{\infty }\left(C_{n}e^{i\lambda_{n}L}+D_{n}e^{-i\lambda_{n}L}\right)P_{mn},$$ (30)

$$B_{m}=-\delta_{m0}+\frac{1}{O_{m}}\sum_{n=0}^{\infty }\left(C_{n}e^{-i\lambda_{n}L}+D_{n}e^{i\lambda_{n}L}\right)Q_{mn},$$ (31)

$$F_{m}=\frac{1}{O_{m}}\sum_{n=0}^{\infty }\left(C_{n}e^{i\lambda_{n}L}+D_{n}e^{-i\lambda_{n}L}\right)Q_{mn},$$ (32)

where the modal coupling integrals which involves the eigenfunctions of rigid and flexible regions are defined by

$$P_{mn}=\int_{0}^{a}R_{1}(\tau_{m},r)R_{3}(\xi_{n},r)r\;dr,\;Q_{mn}=\int_{a}^{b}R_{2}(\gamma_{m},r)R_{3}(\xi_{n},r)r\;dr.$$ (33)

To facilitate further analysis, symmetric and antisymmetric combinations of the coefficients are defined. Adding and subtracting Eqs (29) and (30) yields:

$$\Psi^{+}=A_{m}+E_{m}=-\delta_{m0}+\frac{2}{M_{m}}\sum_{n=0}^{\infty }\Delta^{+}\cos(\lambda_{n}L)P_{mn},$$ (34)

$$\Psi^{-}=A_{m}-E_{m}=-\delta_{m0}-\frac{2i}{M_{m}}\sum_{n=0}^{\infty }\Delta^{-}\sin(\lambda_{n}L)P_{mn},$$ (35)

and similarly, from Eqs (31) and (32):

$$\Xi^{+}=B_{m}+F_{m}=-\delta_{m0}+\frac{2}{O_{m}}\sum_{n=0}^{\infty }\Delta^{+}\cos(\lambda_{n}L)Q_{mn},$$ (36)

$$\Xi^{-}=B_{m}-F_{m}=-\delta_{m0}-\frac{2i}{O_{m}}\sum_{n=0}^{\infty }\Delta^{-}\sin(\lambda_{n}L)Q_{mn},$$ (37)

where the symmetric and antisymmetric modal terms are defined as:

$$A_{m}\pm E_{m}=\Psi^{\pm },\;B_{m}\pm F_{m}=\Xi^{\pm },\;C_{m}\pm D_{m}=\Delta^{\pm }.$$ (38)

## 4 Galerkin formulation

We enforce the continuity of normal velocities at the interfaces z=±L. The Galerkin formulation presented in [15] is employed to simulate the vibrational response of membranes connected along z=±L, for *b* < *r* < *d*. The dimensionless equations of motion for the elastic membrane discs are

$$\frac{\partial^{2}q_{1}}{\partial r^{2}}+\mu^{2}q_{1}=\alpha_{M}\phi_{3},\;z=-L,\;b<r<d,$$ (39)

$$\frac{\partial^{2}q_{2}}{\partial r^{2}}+\mu^{2}q_{2}=-\alpha_{M}\phi_{3},\;z=L,\;b<r<d,$$ (40)

where $\mu_{M}=c/c_{m}$ and $\alpha_{M}=\rho c^{2}/(Tk)$ denote the dimensionless membrane wave number and the fluid loading parameter, respectively. The functions *q*_1_(*r*) and *q*_2_(*r*) represent the dimensionless membrane displacements at *z* = −*L* and *z* = *L*.

### Spring-like edge conditions at *r* = *b.*

The spring-like edge conditions at *r* = *b* with coupling constant $\sigma_{1}=k{\bar{\sigma }}_{1}$ are

$$\sigma_{1}q_{1}+\frac{\partial q_{1}}{\partial r}=0,\;z=-L,$$ (41)

$$\sigma_{1}q_{2}+\frac{\partial q_{2}}{\partial r}=0,\;z=L.$$ (42)

### Spring-like edge conditions at *r* = *d.*

Similarly, at *r* = *d* with coupling constant $\sigma_{2}=k{\bar{\sigma }}_{2}$, we have

$$\sigma_{2}q_{1}+\frac{\partial q_{1}}{\partial r}=0,\;z=-L,$$ (43)

$$\sigma_{2}q_{2}+\frac{\partial q_{2}}{\partial r}=0,\;z=L.$$ (44)

The displacements *q*_1_(*r*) and *q*_2_(*r*) are expressed using Fourier series:

$$q_{1}(r)=\sum_{n=0}^{\infty }g_{1n}R_{n}(r),$$ (45)

$$q_{2}(r)=\sum_{n=0}^{\infty }g_{2n}R_{n}(r),$$ (46)

where *g*_1*n*_ and *g*_2*n*_ (n=0,1,2,…) are unknown coefficients and *R*_*n*_(*r*) is the *n*-th eigenfunction satisfying

$$\frac{d^{2}R_{n}}{dr^{2}}+\varrho_{n}^{2}R_{n}(r)=0,\;z=\pm L,\;b<r<d.$$ (47)

The associated boundary conditions are

$$\sigma_{1}R_{n}(b)+R_{n}^{\prime }(b)=0,$$ (48)

$$\sigma_{2}R_{n}(d)+R_{n}^{\prime }(d)=0.$$ (49)

Solving (47) with (48) and (49) yields


$$R_{n}(r)=\sigma_{1}\sin[\varrho_{n}(r-b)]-\varrho_{n}\cos[\varrho_{n}(r-b)],$$

where the eigenvalues $\varrho_{n}$ are roots of the characteristic equation

$$(\sigma_{1}\sigma_{2}+\varrho_{n}^{2})\sin[\varrho_{n}(d-b)]+(\sigma_{1}-\sigma_{2})\varrho_{n}\cos[\varrho_{n}(d-b)]=0,\;n=0,1,2,\ldots .$$ (50)

These roots can be computed numerically; their properties are discussed in [22]. The eigenfunctions *R*_*n*_ satisfy the orthogonality relation

$$\int_{b}^{d}R_{n}(r)R_{m}(r)\;dr=\delta_{mn}H_{n},$$ (51)

where

$$H_{n}=\int_{b}^{d}R_{n}^{2}(r)\;dr.$$ (52)

To determine *g*_1*n*_ and *g*_2*n*_, we substitute (9), (45), and (46) into (39) and (40), leading to

$$\sum_{n=0}^{\infty }g_{1n}(\mu^{2}-\varrho_{n}^{2})R_{n}(r)=\alpha_{M}\sum_{n=0}^{\infty }\left(C_{n}e^{-i\lambda_{n}L}+D_{n}e^{i\lambda_{n}L}\right)R_{3}(\xi_{n},r),$$ (53)

$$\sum_{n=0}^{\infty }g_{2n}(\mu^{2}-\varrho_{n}^{2})R_{n}(r)=-\alpha_{M}\sum_{n=0}^{\infty }\left(C_{n}e^{i\lambda_{n}L}+D_{n}e^{-i\lambda_{n}L}\right)R_{3}(\xi_{n},r).$$ (54)

Multiplying both sides by *R*_*m*_(*r*) and integrating over [*b*,*d*], using (51), we obtain

$$g_{1m}=\frac{\alpha_{M}}{H_{m}(\mu^{2}-\varrho_{m}^{2})}\sum_{n=0}^{\infty }\left(C_{n}e^{-i\lambda_{n}L}+D_{n}e^{i\lambda_{n}L}\right)L_{mn},$$ (55)

$$g_{2m}=-\frac{\alpha_{M}}{H_{m}(\mu^{2}-\varrho_{m}^{2})}\sum_{n=0}^{\infty }\left(C_{n}e^{i\lambda_{n}L}+D_{n}e^{-i\lambda_{n}L}\right)L_{mn},$$ (56)

where

$$L_{mn}=\int_{b}^{d}R_{m}(r)R_{3}(\xi_{n},r)\;dr.$$ (57)

Adding and subtracting (55) and (56) gives

$$g_{1m}+g_{2m}=-\frac{2i\alpha_{M}}{H_{m}(\mu^{2}-\varrho_{m}^{2})}\sum_{n=0}^{\infty }\Delta_{n}^{-}\sin(\lambda_{n}L)L_{mn},$$ (58)

$$g_{1m}-g_{2m}=\frac{2\alpha_{M}}{H_{m}(\mu^{2}-\varrho_{m}^{2})}\sum_{n=0}^{\infty }\Delta_{n}^{+}\cos(\lambda_{n}L)L_{mn}.$$ (59)

The normal velocity continuity conditions at z=±L can be expressed as:

$$\frac{\partial \phi_{3}}{\partial z}(r,-L)=\left\{ \begin{array}{cc} \frac{\partial \phi_{1}}{\partial z}(r,-L), & 0<r<a,\\ \frac{\partial \phi_{2}}{\partial z}(r,-L), & a<r<b,\\ q_{1}(r), & b<r<d, \end{array}\right.$$ (60)

$$\frac{\partial \phi_{3}}{\partial z}(r,L)=\left\{ \begin{array}{cc} \frac{\partial \phi_{4}}{\partial z}(r,L), & 0<r<a,\\ \frac{\partial \phi_{5}}{\partial z}(r,L), & a<r<b,\\ q_{2}(r), & b<r<d. \end{array}\right.$$ (61)

Multiplying Eqs (60) and (61) by $R_{3}(\xi_{m},r)r$ and integrating from *r* = 0 to *r* = *d*, we obtain:

$$\int_{0}^{d}\phi_{3z}(r,-L)R_{3}(\xi_{m},r)r\;dr=\int_{0}^{a}\phi_{1z}(r,-L)R_{3}(\xi_{m},r)r\;dr\;+\int_{a}^{b}\phi_{2z}(r,-L)R_{3}(\xi_{m},r)r\;dr\;+\int_{b}^{d}q_{1}(r)R_{3}(\xi_{m},r)r\;dr,$$ (62)

$$\int_{0}^{d}\phi_{3z}(r,L)R_{3}(\xi_{m},r)r\;dr=\int_{0}^{a}\phi_{4z}(r,L)R_{3}(\xi_{m},r)r\;dr\;+\int_{a}^{b}\phi_{5z}(r,L)R_{3}(\xi_{m},r)r\;dr\;+\int_{b}^{d}q_{2}(r)R_{3}(\xi_{m},r)r\;dr.$$ (63)

Substituting Eqs (9)–(13) and (45)–(46) into (62) and (63), and simplifying, we obtain:

$$C_{m}e^{-i\lambda_{m}L}-D_{m}e^{i\lambda_{m}L}=\frac{R_{3}^{\prime }(\xi_{m},d)}{\lambda_{m}X_{m}}\left(\frac{\Pi_{0}}{\lambda_{m}^{2}-\beta^{2}}+(2-\xi_{m}^{2})\Pi_{1}-\Pi_{2}\right)\;+\frac{\alpha \Omega_{1m}}{d\lambda_{m}X_{m}}+\frac{\alpha }{id\lambda_{m}X_{m}}\sum_{n=0}^{\infty }g_{1n}M_{mn},$$ (64)

$$C_{m}e^{i\lambda_{m}L}-D_{m}e^{-i\lambda_{m}L}=\frac{R_{3}^{\prime }(\xi_{m},d)}{\lambda_{m}X_{m}}\left(\frac{\Pi_{3}}{\lambda_{m}^{2}-\beta^{2}}+(2-\xi_{m}^{2})\Pi_{4}-\Pi_{5}\right)\;+\frac{\alpha \Omega_{2m}}{d\lambda_{m}X_{m}}+\frac{\alpha }{id\lambda_{m}X_{m}}\sum_{n=0}^{\infty }g_{2n}M_{mn},$$ (65)

where

$$\Omega_{1m}=P_{0m}+Q_{0m}-\sum_{n=0}^{\infty }\left(A_{n}\eta_{n}P_{nm}+B_{n}s_{n}Q_{nm}\right),$$ (66)

$$\Omega_{2m}=\sum_{n=0}^{\infty }\left(E_{n}\eta_{n}P_{nm}+F_{n}s_{n}Q_{nm}\right),$$ (67)

$$\Pi_{0}=\Gamma \nu^{2}\beta^{2}\sum_{n=0}^{\infty }\frac{\lambda_{n}R_{3}^{\prime }(\xi_{n},d)}{\lambda_{n}^{2}-\beta^{2}}\left(C_{n}e^{-i\lambda_{n}L}-D_{n}e^{i\lambda_{n}L}\right),$$ (68)

$$\Pi_{1}=\sum_{n=0}^{\infty }\lambda_{n}R_{3}^{\prime }(\xi_{n},d)\left(C_{n}e^{-i\lambda_{n}L}-D_{n}e^{i\lambda_{n}L}\right),$$ (69)

$$\Pi_{2}=\sum_{n=0}^{\infty }\lambda_{n}\xi_{n}^{2}R_{3}^{\prime }(\xi_{n},d)\left(C_{n}e^{-i\lambda_{n}L}-D_{n}e^{i\lambda_{n}L}\right),$$ (70)

$$\Pi_{3}=\Gamma \nu^{2}\beta^{2}\sum_{n=0}^{\infty }\frac{\lambda_{n}R_{3}^{\prime }(\xi_{n},d)}{\lambda_{n}^{2}-\beta^{2}}\left(C_{n}e^{i\lambda_{n}L}-D_{n}e^{-i\lambda_{n}L}\right),$$ (71)

$$\Pi_{4}=\sum_{n=0}^{\infty }\lambda_{n}R_{3}^{\prime }(\xi_{n},d)\left(C_{n}e^{i\lambda_{n}L}-D_{n}e^{-i\lambda_{n}L}\right),$$ (72)

$$\Pi_{5}=\sum_{n=0}^{\infty }\lambda_{n}\xi_{n}^{2}R_{3}^{\prime }(\xi_{n},d)\left(C_{n}e^{-i\lambda_{n}L}-D_{n}e^{i\lambda_{n}L}\right),$$ (73)

$$M_{mn}=\int_{b}^{d}R_{n}(r)R_{3}(\xi_{m},r)r\;dr.$$ (74)

Adding and subtracting Eqs (64) and (65) yields:

$$\Delta_{m}^{-}=\frac{R_{3}^{\prime }(\xi_{m},d)}{2\lambda_{m}\cos(\lambda_{m}L)X_{m}}\left(\frac{\Pi_{03}^{+}}{\lambda_{m}^{2}-\beta^{2}}+(2-\xi_{m}^{2})\Pi_{14}^{+}-\Pi_{25}^{+}\right)\;+\frac{\alpha \Omega_{12m}^{+}}{2d\lambda_{m}\cos(\lambda_{m}L)X_{m}}+\frac{\alpha }{2id\lambda_{m}\cos(\lambda_{m}L)X_{m}}\sum_{n=0}^{\infty }(g_{1n}+g_{2n})M_{mn},$$ (75)

$$\Delta_{m}^{+}=-\frac{R_{3}^{\prime }(\xi_{m},d)}{2i\lambda_{m}\sin(\lambda_{m}L)X_{m}}\left(\frac{\Pi_{03}^{-}}{\lambda_{m}^{2}-\beta^{2}}+(2-\xi_{m}^{2})\Pi_{14}^{-}-\Pi_{25}^{-}\right)\;-\frac{\alpha \Omega_{12m}^{-}}{2id\lambda_{m}\sin(\lambda_{m}L)X_{m}}+\frac{\alpha }{2d\lambda_{m}\sin(\lambda_{m}L)X_{m}}\sum_{n=0}^{\infty }(g_{1n}-g_{2n})M_{mn},$$ (76)

where


$$\Pi_{03}^{\pm }=\Pi_{0}\pm \Pi_{3},\;\Pi_{14}^{\pm }=\Pi_{1}\pm \Pi_{4},\;\Pi_{25}^{\pm }=\Pi_{2}\pm \Pi_{5},$$

$$\Omega_{12m}^{\pm }=\Omega_{1m}\pm \Omega_{2m}=P_{0m}+Q_{0m}-\sum_{n=0}^{\infty }\left(\Psi_{n}^{\mp }\eta_{n}P_{nm}+\Xi_{n}^{\mp }s_{n}Q_{nm}\right).$$

For clamped edge conditions, we have $\Pi_{0}=\Pi_{1}=\Pi_{3}=\Pi_{4}=0$, which implies $\Pi_{03}^{\pm }=\Pi_{14}^{\pm }=0$. The remaining terms $\Pi_{25}^{\pm }$ can be determined using the boundary condition $\partial \phi_{3}/\partial r=0$ at z=±L, which leads to:

$$\sum_{m=0}^{\infty }\Delta_{m}^{+}\cos(\lambda_{m}L)R_{3}^{\prime }(\xi_{n},d)=0,$$ (77)

$$\sum_{m=0}^{\infty }\Delta_{m}^{-}\sin(\lambda_{m}L)R_{3}^{\prime }(\xi_{n},d)=0.$$ (78)

Multiplying Eq (75) by $\sin(\lambda_{m}L)R_{3}^{\prime }(\xi_{n},d)$, summing over *m* and using (78), we obtain:

$$\Pi_{25}^{+}=\frac{\alpha }{2S_{25}^{+}d}\sum_{m=0}^{\infty }\frac{\sin(\lambda_{m}L)R_{3}^{\prime }(\xi_{n},d)\Omega_{12m}^{+}}{\lambda_{m}\cos(\lambda_{m}L)X_{m}}\;+\frac{\alpha }{2S_{25}^{+}d}\sum_{m=0}^{\infty }\frac{\sin(\lambda_{m}L)R_{3}^{\prime }(\xi_{n},d)}{i\lambda_{m}\cos(\lambda_{m}L)X_{m}}\sum_{n=0}^{\infty }(g_{1n}+g_{2n})M_{mn},$$ (79)

$$\Pi_{25}^{-}=-\frac{\alpha }{2S_{25}^{-}d}\sum_{m=0}^{\infty }\frac{\cos(\lambda_{m}L)R_{3}^{\prime }(\xi_{n},d)\Omega_{12m}^{-}}{i\lambda_{m}\sin(\lambda_{m}L)X_{m}}\;+\frac{\alpha }{2S_{25}^{-}d}\sum_{m=0}^{\infty }\frac{\cos(\lambda_{m}L)R_{3}^{\prime }(\xi_{n},d)}{\lambda_{m}\sin(\lambda_{m}L)X_{m}}\sum_{n=0}^{\infty }(g_{1n}-g_{2n})M_{mn},$$ (80)

where


$$S_{25}^{+}=\sum_{m=0}^{\infty }\frac{\sin(\lambda_{m}L)(R_{3}^{\prime }(\xi_{m},d){)}^{2}}{2i\lambda_{m}\cos(\lambda_{m}L)X_{m}},\;S_{25}^{-}=\sum_{m=0}^{\infty }\frac{\cos(\lambda_{m}L)(R_{3}^{\prime }(\xi_{m},d){)}^{2}}{2\lambda_{m}\sin(\lambda_{m}L)X_{m}}.$$

## 5 Numerical results and discussions

Upon defining the analytical parameters and truncating the resulting system of linear equations to *N* terms, the solution is obtained numerically. To investigate the physical behavior, the incident, reflected, and transmitted fields in regions I, II, IV, and V are used to derive the corresponding acoustic power expressions within the duct sections. The fundamental modes radiating from regions I and II yield incident power values of $\varepsilon_{\text{I}}^{i}=a^{2}/2$ and $\varepsilon_{\text{II}}^{i}=(b^{2}$ − *a*^2^)/2, respectively. Furthermore, the scattered power components at the inlet and outlet are expressed as:

$$\varepsilon_{\text{I}}^{r}=-\text{Re}\left[\sum_{n=0}^{\infty }|B_{n}{|}^{2}\eta_{n}M_{n}\right],\;\;\varepsilon_{\text{II}}^{r}=-\text{Re}\left[\sum_{n=0}^{\infty }|D_{n}{|}^{2}s_{n}O_{n}\right],$$ (81)

$$\varepsilon_{\text{IV}}^{t}=\text{Re}\left[\sum_{n=0}^{\infty }|G_{n}{|}^{2}\eta_{n}M_{n}\right],\;\;\varepsilon_{\text{V}}^{t}=\text{Re}\left[\sum_{n=0}^{\infty }|I_{n}{|}^{2}s_{n}O_{n}\right].$$ (82)

The negative sign in Eq (81) signifies that the reflected energy travels in the –*z* direction. By invoking energy conservation, the total incident power must equal the total scattered and transmitted powers, leading to the following identity:

$$\varepsilon_{\text{I}}^{i}+\varepsilon_{\text{II}}^{i}=\varepsilon_{\text{I}}^{r}+\varepsilon_{\text{II}}^{r}+\varepsilon_{\text{IV}}^{t}+\varepsilon_{\text{V}}^{t}.$$ (83)

For numerical implementation, this relation is normalized by dividing both sides by the sum of the incident powers, yielding:

$$1=\varepsilon_{1}+\varepsilon_{2}+\varepsilon_{4}+\varepsilon_{5},$$ (84)

where the normalized quantities are defined as follows: $\varepsilon_{1}=\varepsilon_{\text{I}}^{r}/(\varepsilon_{\text{I}}^{i}$  +  $\varepsilon_{\text{II}}^{i})$, $\varepsilon_{2}=\varepsilon_{\text{II}}^{r}/(\varepsilon_{\text{I}}^{i}$  +  $\varepsilon_{\text{II}}^{i})$, $\varepsilon_{4}=\varepsilon_{\text{IV}}^{t}/(\varepsilon_{\text{I}}^{i}$  +  $\varepsilon_{\text{II}}^{i})$, and $\varepsilon_{5}=\varepsilon_{\text{V}}^{t}/(\varepsilon_{\text{I}}^{i}$  +  $\varepsilon_{\text{II}}^{i})$. To perform the numerical evaluation, fixed values are assigned to the involving physical parameters. The elastic shell under study is made of aluminum, featuring a thickness of *h* = 0.002 m and a material density of $\rho_{s}=2700$ kg⋅m^−3^. The material properties are defined by a Poisson’s ratio of ν=0.34 and a Young’s modulus of $E=7.2\times 10^{10}$ N⋅m^−2^. The enclosed fluid medium is air, considered compressible, with a density of ρ=1.2043 kg⋅m^−3^ and a speed of sound *c* = 343 m/s. The membrane disc is assumed to be stainless steel, with an areal mass density $\rho_{m}=0.1715$ kg⋅m^−2^ and pre-applied tension *T* = 350 N. Both stretching coefficients are set as $\sigma_{1}=\sigma_{2}=1$. The experimental values of the aforementioned parameters and their ranges are given in [28] and has been used in literature, for instance [13,16,29–31]. Note that we employed *Mathematica* with built-in commands, such as FindRoot and NSolve, to numerically find roots and solve the truncated systems, respectively. Figures were generated using standard plotting functions, including Plot and ListPlot. To assess the performance of the configuration as a reactive acoustic component, the transmission loss (TL) is defined by:

$$\text{TL}=-10\log_{10}[\varepsilon_{4}+\varepsilon_{5}].$$ (85)

### 5.1 Validation of truncated solution

**Fig 2:**
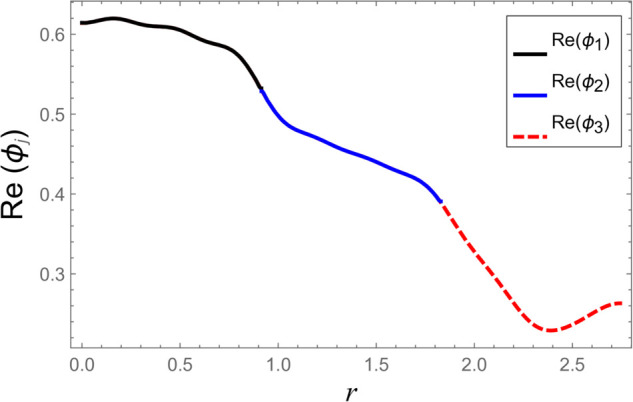
At interfaces *z* = −*L*: Real parts of pressures vs. duct radius with frequency 500Hz and truncation parameter *N* = 60 terms.

**Fig 3:**
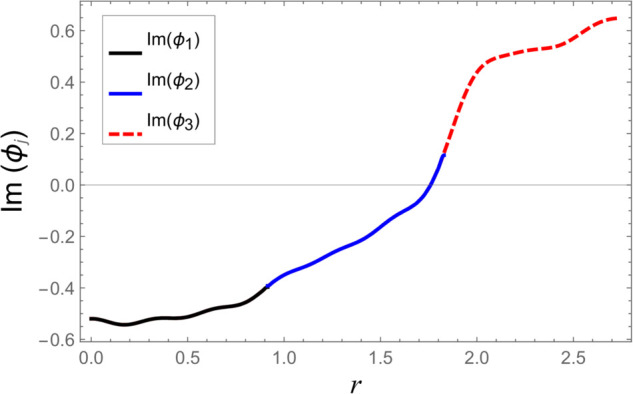
At interfaces *z* = −*L*: Imaginary parts of pressures vs. duct radius with frequency 500Hz and truncation parameter *N* = 60 terms.

**Fig 4:**
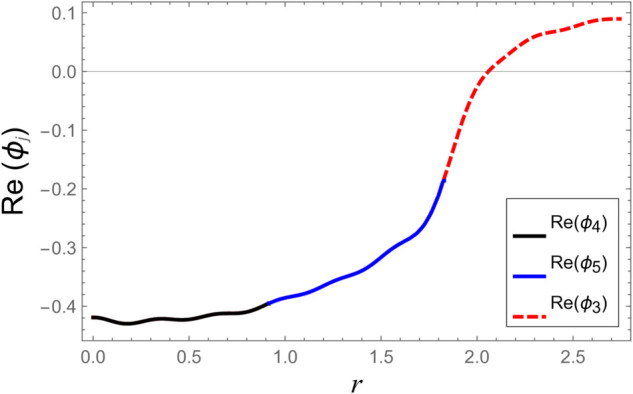
At interfaces *z* = *L*: Real parts of pressures vs. duct radius with frequency 500Hz and truncation parameter *N* = 60 terms.

**Fig 5:**
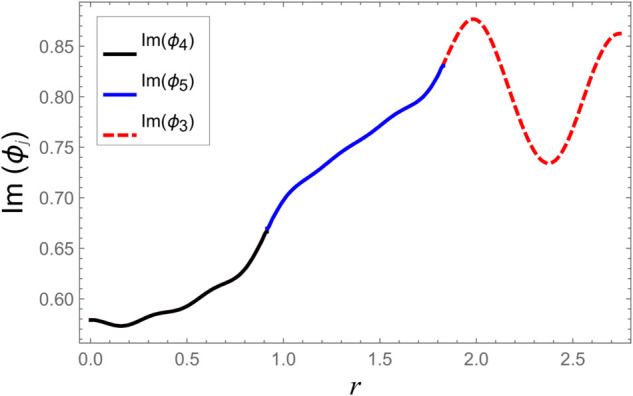
At interfaces *z* = *L*: Imaginary parts of pressures vs. duct radius with frequency 500Hz and truncation parameter *N* = 60 terms.

**Fig 6:**
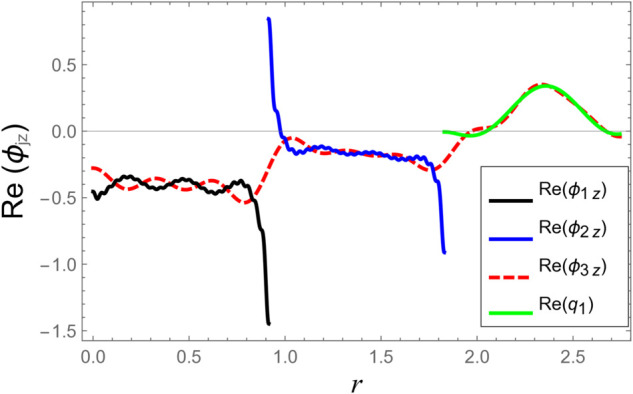
At interface *z* = −*L*: Real parts of normal velocities vs. duct radius with frequency 500Hz and truncation parameter *N* = 60 terms.

**Fig 7:**
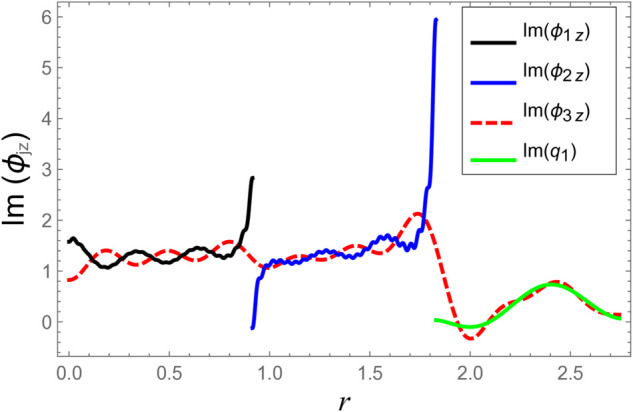
At interface *z* = −*L*: Imaginary parts of normal velocities vs. duct radius with frequency 500Hz and truncation parameter *N* = 60 terms.

**Fig 8:**
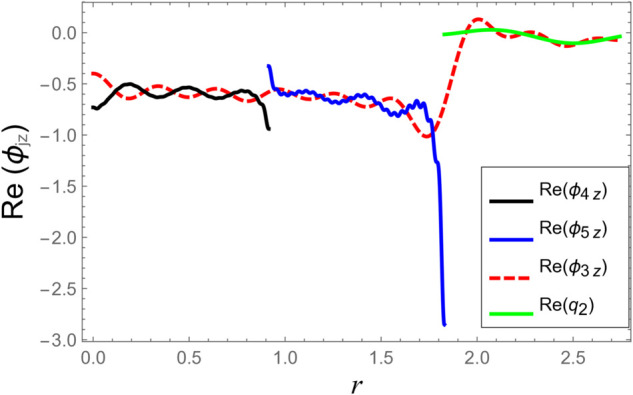
At interface *z* = *L*: Real parts of velocities vs. duct radius with frequency 500Hz and truncation parameter *N* = 60 terms.

**Fig 9:**
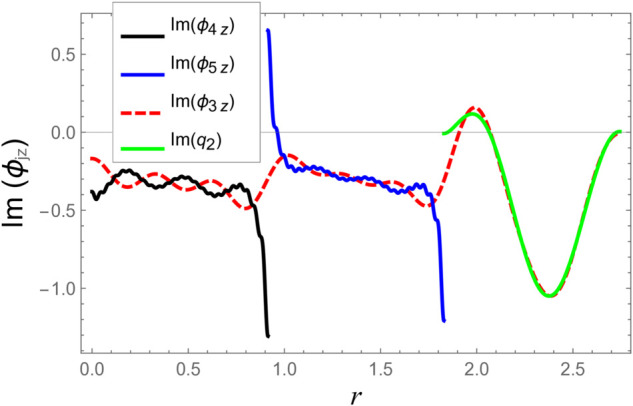
At interface *z* = *L*: Imaginary parts of velocities vs. duct radius with frequency 500Hz and truncation parameter *N* = 60 terms.

To ensure the reliability of the solution approach and validate the numerical accuracy, a reconstruction of the continuity (matching) conditions is carried out. This verification ensures that the chosen number of retained modes leads to solution convergence. The numerical test considers duct radii as $\bar{a}=0.1$ m, $\bar{b}=0.2$ m, and $\bar{d}=0.3$ m. The half-length of the cavity is taken as $\bar{L}=0.1$ m, with an excitation frequency of *f* = 500 Hz. At the interfaces located at z=±L, the continuity of pressure and normal velocity is checked using 60 series terms, retaining 61 modal components. The computed results, illustrated in Figs 2–9, indicate a close match of dimensionless pressure and velocity profiles on either side of the interfaces, thereby confirming the accurate enforcement of the boundary continuity conditions. The real and imaginary components of the pressure at *z* = −*L* are shown in Figs 2 and 3, while those at *z* = *L* are depicted in Figs 4 and 5.

Figs 6–9 present a detailed comparison of the normal velocity distributions across the duct radius at the axial interfaces located at z=±L. Figs 6 and 7 display the real and imaginary parts of the velocity at *z* = −*L*, whereas Figs 8 and 9 present the corresponding results at *z* = *L*. These plots serve to validate the satisfaction of the velocity matching conditions at the junctions between adjacent acoustic regions. The results indicate that the real and imaginary components of the acoustic velocity fields exhibit continuity across the interfaces, confirming the correctness of the mode-matching implementation. In particular, within the chamber region III, where the membrane occupies the annular region bounded by *b* < *r* < *d*, the computed normal velocities of the fluid align closely with the dynamic transverse displacement of the membrane surfaces. This agreement verifies that the fluid–structure interaction is accurately captured, demonstrating that the kinematic continuity condition at the fluid–membrane interface is rigorously enforced in the numerical model.

However, as the radial coordinate *r* approaches the inner and outer junctions at *r* = *a* and *r* = *b*, noticeable edge effects appear in the plotted velocity profiles. These sharp features arise due to the abrupt change in boundary conditions at the chamber ends, where the fluid velocity must transition from a finite value within the fluid domain to zero along the rigid annular portions of the structure. Such abrupt transitions inherently cause localized singularities or boundary layer effects near the corners, which manifest as non-smooth variations in the velocity profiles. These edge singularities are a well-known artifact of idealized boundary conditions in modal or spectral methods and do not represent physical inconsistencies or instabilities in the solution.

Additionally, the velocity profiles exhibit mild oscillations near the transition regions. These fluctuations are attributable to the Gibbs phenomenon, which is a well-known in spectral or modal solutions involving truncated series expansions. When a finite number of modes is used to represent discontinuous or sharply varying fields, oscillatory forms may occur near the points of discontinuity. This phenomenon and its implications for convergence and accuracy in similar acoustic problems are discussed comprehensively in [16].

**Fig 10:**
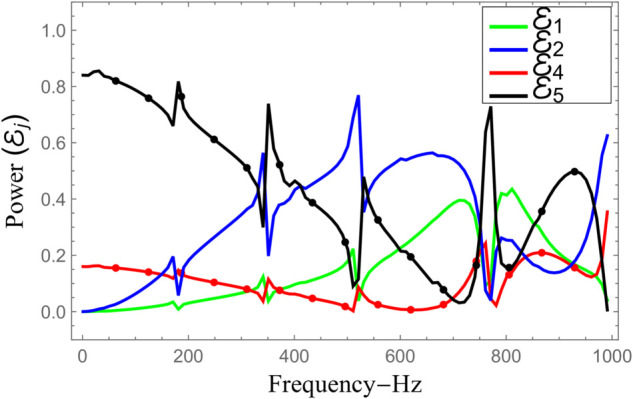
Scattering powers against frequency, where $\bar{a}=0.1$m, $\bar{b}=0.2$m, $\bar{d}=0.28$m, $\bar{L}=0.25$m and *N* = 60 terms.

**Fig 11:**
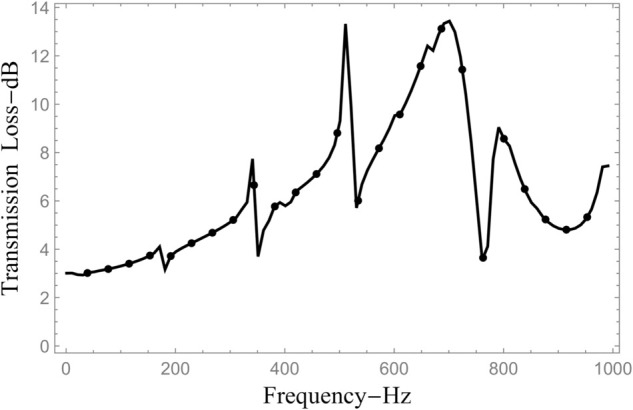
Transmission loss against frequency, where $\bar{a}=0.1$m, $\bar{b}=0.2$m, $\bar{d}=0.28$m, $\bar{L}=0.25$m and *N* = 60 terms.

To investigate the energy conservation and the behaviour of acoustic scattering across the system, numerical evaluations were performed by computing the amplitudes of 61 modal components. These include all propagating modes that contribute significantly to the acoustic field. The configuration is maintained by ensuring that the radii of the inlet and outlet ducts are smaller than the radius of the central expansion chamber. Specifically, the geometric parameters are fixed as follows: $\bar{a}=0.1$ m, $\bar{b}=0.2$ m, $\bar{d}=0.28$ m, and the half-length of the cavity is set to $\bar{L}=0.25$ m. The system is analyzed under clamped boundary conditions while the excitation frequency is varied continuously from 1 Hz to 1000 Hz. Figs 10 and 11 illustrates the frequency-dependent behaviour of power flow and transmission loss (TL) across different regions. In Fig 10, power components are displayed: curves with mesh patterns correspond to transmitted energy into outlet sections IV and V, whereas the smooth curves represent reflected energy in inlet sections I and II. The computed results reveal that energy transmission through the annular domains (regions II and V) is consistently higher than that through the central axial ducts (regions I and IV). At lower frequencies, particularly at the onset, region V conveys more than 80% of the incoming energy. However, this transmission gradually declines with oscillatory variations and reaches a local minimum at approximately 748 Hz, which marks the onset of the next structural (flexural) mode in the chamber, as seen in Fig 10. Interestingly, the reflected power in region II displays an inverse trend relative to the transmitted power in region V, increasing around frequencies where region V experiences energy dips. The central ducts, namely regions I and IV, also exhibit lower energy transfer overall, following a trend opposite to that of the outer annular paths. Despite these spatial variations, the total energy balance remains conserved. Numerical verification confirms that the cumulative reflected and transmitted powers across all regions satisfy the conservation identity described in Eq (84) throughout the entire frequency range, validating the correctness of the scattering formulation. Further insights are provided in Fig 11, which presents the transmission loss curve as a function of frequency. The TL exhibits pronounced peaks at specific frequencies associated with resonant conditions of the flexible shell, with a maximum value of approximately 13 dB near 748 Hz. The stop band centered around this frequency is noticeably broader than those at other frequencies in the analyzed range, indicating enhanced acoustic attenuation due to strong fluid–structure coupling. In addition, several sharp transitions and oscillations appear in the power curves of Figs 10 and 11. These features are attributed to the resonance behavior of the tensioned membrane discs installed at z=±L in the annular region bounded by *b* < *r* < *d*, where the membrane dynamics modify the effective impedance at the junctions and, in turn, influence the overall scattering and transmission characteristics of the structure.

### 5.2 Influence of membrane edge constraints on acoustic scattering behavior

**Fig 12:**
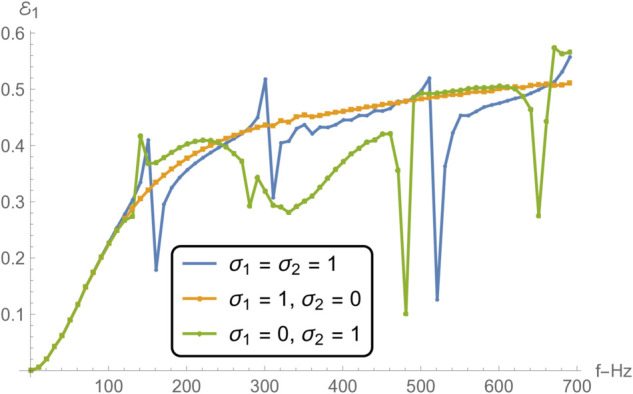
Reflection in region I as a function of frequency for different edge conditions applied to the membrane disc rings at *r* = *b* and *r* = *d*: (i) spring-like constraints at both edges ($\sigma_{1}=1,\sigma_{2}=1$); (ii) a spring-like constraint at *r* = *b* and a free edge at *r* = *d* ($\sigma_{1}=1,\sigma_{2}=0$); and (iii) a free edge at *r* = *b* with a spring-like constraint at *r* = *d* ($\sigma_{1}=0,\sigma_{2}=1$).

Figs 12 and 15 collectively examine how varying boundary conditions at the edges of a membrane disc, located between radii *r* = *b* and *r* = *d*, influence the frequency-dependent acoustic reflection and transmission in adjacent duct regions. The three considered configurations involve: (i) spring-like constraints at both edges ($\sigma_{1}=\sigma_{2}=1$), (ii) a spring-like constraint at *r* = *b* and a free edge at *r* = *d* ($\sigma_{1}=1$, $\sigma_{2}=0$), and (iii) a free edge at *r* = *b* and a spring-like constraint at *r* = *d* ($\sigma_{1}=0$, $\sigma_{2}=1$).

**Fig 13:**
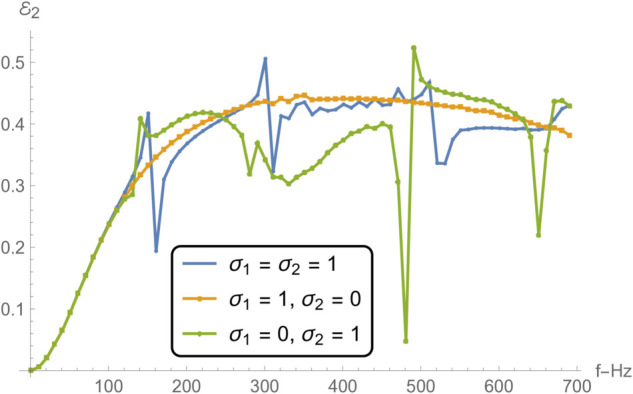
Reflection in region II as a function of frequency for different edge conditions applied to the membrane disc rings at *r* = *b* and *r* = *d*: (i) spring-like constraints at both edges ($\sigma_{1}=1,\sigma_{2}=1$); (ii) a spring-like constraint at *r* = *b* and a free edge at *r* = *d* ($\sigma_{1}=1,\sigma_{2}=0$); and (iii) a free edge at *r* = *b* with a spring-like constraint at *r* = *d* ($\sigma_{1}=0,\sigma_{2}=1$).

**Fig 14:**
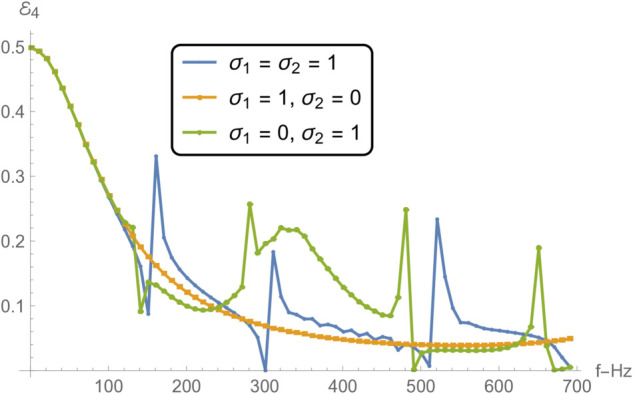
Transmission in region IV as a function of frequency for different edge conditions applied to the membrane disc rings at *r* = *b* and *r* = *d*: (i) spring-like constraints at both edges ($\sigma_{1}=1,\sigma_{2}=1$); (ii) a spring-like constraint at *r* = *b* and a free edge at *r* = *d* ($\sigma_{1}=1,\sigma_{2}=0$); and (iii) a free edge at *r* = *b* with a spring-like constraint at *r* = *d* ($\sigma_{1}=0,\sigma_{2}=1$).

In Figs 12 and 13 , which respectively present the reflected acoustic power in regions I and II, all cases show clear spectral oscillations induced by resonances of the membrane structure. The symmetric spring–spring configuration (case i) yields the smoothest reflection profiles with relatively moderate amplitude variations, indicative of improved impedance continuity and reduced scattering. In contrast, asymmetric edge constraints, particularly the spring–free configuration (case ii)—lead to sharper reflection peaks, especially near resonant frequencies, where local structural compliance enhances the membrane–fluid interaction. The free–spring configuration (case iii) exhibits an intermediate response with moderately elevated reflections at lower frequencies. This difference in reflection characteristics can be attributed to the variation in the membrane’s effective stiffness and mass loading distribution at its boundaries, which alters its modal shapes and resonance amplitudes. In particular, free edges enable larger local displacements at the membrane rim, intensifying the coupling between the membrane and the adjacent acoustic field.

**Fig 15:**
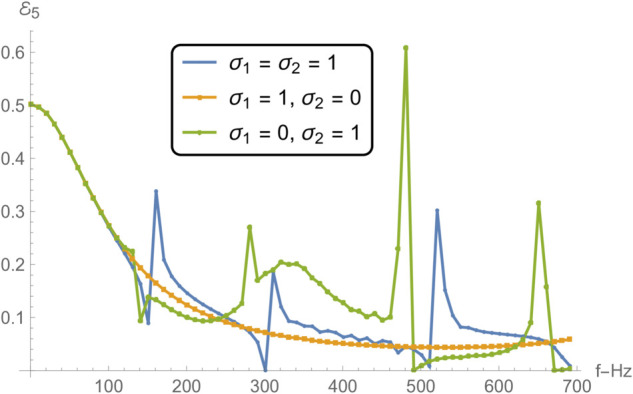
Transmission in V as a function of frequency for different edge conditions applied to the membrane disc rings at *r* = *b* and *r* = *d*: (i) spring-like constraints at both edges ($\sigma_{1}=1,\sigma_{2}=1$); (ii) a spring-like constraint at *r* = *b* and a free edge at *r* = *d* ($\sigma_{1}=1,\sigma_{2}=0$); and (iii) a free edge at *r* = *b* with a spring-like constraint at *r* = *d* ($\sigma_{1}=0,\sigma_{2}=1$).

Figs 14 and 15, showing the transmitted acoustic power in regions IV and V respectively, complement the reflection results by highlighting how energy propagates through the membrane under the same boundary conditions. The spring–spring configuration again facilitates broadband, relatively uniform transmission, suggesting that symmetrical stiffness promotes smoother wave passage. The presence of a free edge, as in case ii, significantly alters the transmission spectrum, giving rise to pronounced peaks and troughs due to strong modal excitation and local displacement amplification. Case iii yields intermediate behavior, with higher transmission at lower frequencies but more variability near resonance bands. These transmission variations are linked to the constructive and destructive interference between the incident and membrane-radiated acoustic fields, whose relative phases depend on the vibration mode excited and the spatial distribution of membrane motion.

Notably, the locations of the transmission peaks and reflection dips correspond to the natural frequencies of the membrane-disc structure, revealing constructive and destructive interference patterns arising from strong structure–acoustic coupling. From a structural dynamics perspective, these natural frequencies are determined by the combined effects of membrane tension, geometry, and boundary stiffness, and can be estimated from analytical dispersion relations for axisymmetric membrane modes. The sharpest spectral features occur when the excitation frequency matches a mode whose displacement profile couples strongly with the surrounding duct modes. These resonant features are especially prominent under configurations involving free boundaries, which allow greater membrane displacement and more intense acoustic scattering.

Together, Figs 7 and 8 demonstrate that membrane edge constraints serve as a powerful tuning mechanism for controlling acoustic energy distribution in duct-based systems. By selectively modifying the boundary stiffness, one can manipulate both reflection and transmission characteristics across the frequency spectrum. This capability is particularly relevant in engineering contexts such as adaptive silencers, reconfigurable acoustic filters, and duct liners, where precise control over scattering and transmission is required to meet performance targets. This insight is valuable for the design of tunable acoustic elements, including filters, absorbers, and liners, where tailored scattering properties are essential.

### 5.3 Radius-dependent scattering behavior under varying membrane edge constraints

**Fig 16:**
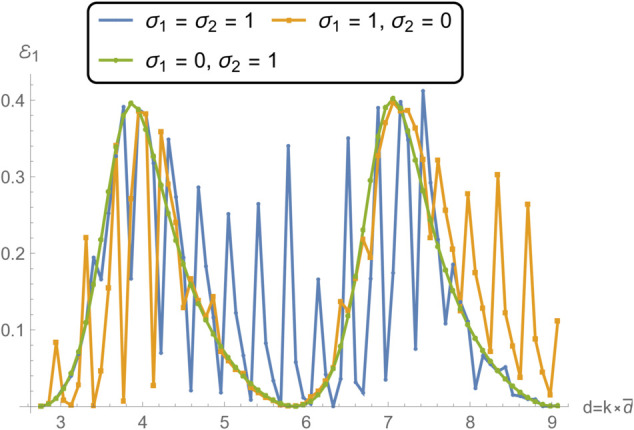
Reflection in region I as a function radius d for different edge conditions applied to the membrane disc rings at *r* = *b* and *r* = *d*: (i) spring-like constraints at both edges ($\sigma_{1}=1,\sigma_{2}=1$); (ii) a spring-like constraint at *r* = *b* and a free edge at *r* = *d* ($\sigma_{1}=1,\sigma_{2}=0$); and (iii) a free edge at *r* = *b* with a spring-like constraint at *r* = *d* ($\sigma_{1}=0,\sigma_{2}=1$).

**Fig 17:**
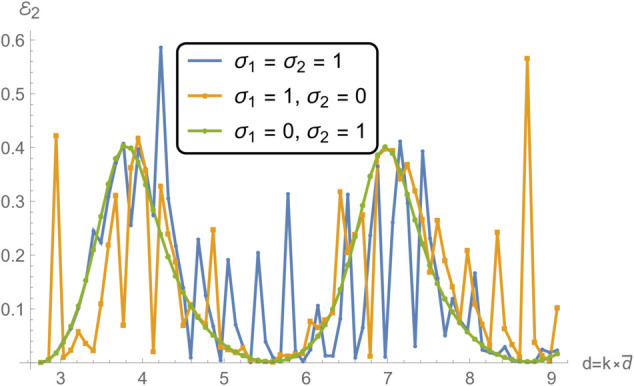
Reflection in region II as a function radius d for different edge conditions applied to the membrane disc rings at *r* = *b* and *r* = *d*: (i) spring-like constraints at both edges ($\sigma_{1}=1,\sigma_{2}=1$); (ii) a spring-like constraint at *r* = *b* and a free edge at *r* = *d* ($\sigma_{1}=1,\sigma_{2}=0$); and (iii) a free edge at *r* = *b* with a spring-like constraint at *r* = *d* ($\sigma_{1}=0,\sigma_{2}=1$).

**Fig 18:**
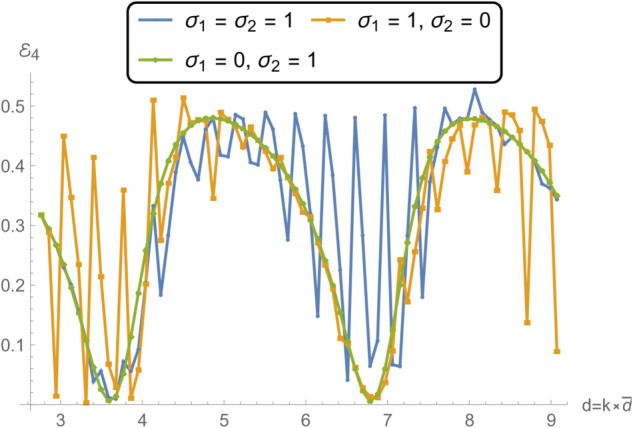
Transmission in region IV as a function radius d for different edge conditions applied to the membrane disc rings at *r* = *b* and *r* = *d*: (i) spring-like constraints at both edges ($\sigma_{1}=1,\sigma_{2}=1$); (ii) a spring-like constraint at *r* = *b* and a free edge at *r* = *d* ($\sigma_{1}=1,\sigma_{2}=0$); and (iii) a free edge at *r* = *b* with a spring-like constraint at *r* = *d* ($\sigma_{1}=0,\sigma_{2}=1$).

**Fig 19:**
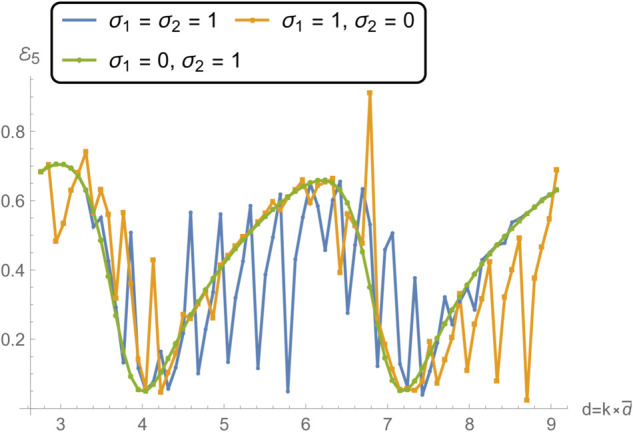
Transmission in region V as a function radius d for different edge conditions applied to the membrane disc rings at *r* = *b* and *r* = *d*: (i) spring-like constraints at both edges ($\sigma_{1}=1,\sigma_{2}=1$); (ii) a spring-like constraint at *r* = *b* and a free edge at *r* = *d* ($\sigma_{1}=1,\sigma_{2}=0$); and (iii) a free edge at *r* = *b* with a spring-like constraint at *r* = *d* ($\sigma_{1}=0,\sigma_{2}=1$).

Figs 16–19 provide further insight into the scattering characteristics of a membrane-disc system embedded in a duct by examining the variation of reflected and transmitted acoustic powers with respect to the outer membrane radius *d*, while keeping the inner radius *b* fixed. The study considers three edge configurations, previously defined as: (i) spring-like constraints at both edges ($\sigma_{1}=\sigma_{2}=1$), (ii) spring-like at *r* = *b* and free at *r* = *d* ($\sigma_{1}=1$, $\sigma_{2}=0$), and (iii) free at *r* = *b* and spring-like at *r* = *d* ($\sigma_{1}=0$, $\sigma_{2}=1$). The figures illustrate how the spatial extent of the membrane influences scattering behavior due to changing modal participation and wave interaction length, as well as the shifting of structural resonances toward lower frequencies as *d* increases, thereby altering the coupling between the acoustic and structural subsystems.

Figs 16 and 17, depicting reflection powers in regions I and II, reveals that increasing the radius *d* generally leads to higher spatial modal density, which intensifies oscillations in the reflection profiles. Across both subplots, the spring–free configuration (case ii) consistently produces larger reflection amplitudes and sharper fluctuations with increasing *d*. This behavior is attributed to enhanced structural flexibility at the free edge, which permits stronger vibrational responses and more pronounced acoustic impedance mismatches at the fluid–structure interfaces. The larger deflection capability of the free edge not only amplifies local pressure fluctuations but also increases the phase variation across the membrane, which reinforces certain reflected wave components through constructive interference. In contrast, the spring–spring case (i) exhibits comparatively smooth variations with lower reflection amplitudes over the same range of *d*, indicating improved energy absorption and reduced scattering due to the symmetric constraint. This symmetric stiffness distribution suppresses asymmetric mode shapes and limits modal conversion, resulting in a more uniform impedance profile. The free–spring configuration (case iii) shows an intermediate trend: while the fluctuations are more prominent than in case (i), they are less severe than in case (ii), particularly at larger values of *d*, where the influence of the free inner edge becomes more pronounced.

Figs 18 and 19, presenting transmission powers in regions IV and V, complements the reflection analysis by illustrating how the expanding membrane disc radius modulates acoustic energy passage through the structure. As *d* increases, the system supports more structural modes, leading to intricate transmission patterns marked by alternating enhancement and suppression of energy transport. Case (ii), involving a free outer edge, continues to exhibit the strongest modal activity and the widest range of transmission variability. The free boundary allows increased displacement near the perimeter, which facilitates stronger interaction with the acoustic field, resulting in higher transmission peaks interleaved with deep nulls due to phase cancellation or anti-resonance effects. These anti-resonances occur when the membrane motion is out of phase with the incident acoustic wave, effectively creating a local cancellation of pressure fluctuations at the interface. The spring–spring case again demonstrates the most stable transmission behavior, with modest amplitude variation and smoother spectral trends, owing to the suppression of excessive membrane vibration. By maintaining balanced edge stiffness, this configuration minimizes the generation of higher-order structural modes that tend to create irregular transmission spectra. The free–spring configuration (case iii) reveals moderate sensitivity to radius variation, especially in region IV, where the influence of the more compliant inner edge becomes increasingly relevant as *d* grows.

Taken together, Figs 16–19 underscore the critical influence of the membrane’s radial extent and edge stiffness on its scattering behavior. The dependence on radius reflects the shifting resonance conditions and mode shapes within the membrane, which directly affect how acoustic energy is reflected and transmitted. Quantitatively, the observed trends are consistent with the scaling of the membrane’s natural frequencies with the inverse of its characteristic length, $f_{n}\propto 1/d$, implying that larger membranes introduce more closely spaced resonances within the frequency band of interest. These results reinforce the idea that structural boundary design and geometric tuning, such as varying *d*, offer effective tools for controlling acoustic performance in waveguide systems. The findings are especially pertinent for applications in which broadband control over wave propagation is required, such as tunable liners, metamaterial absorbers, and smart duct acoustic devices, where design flexibility allows targeted modification of the transmission spectrum to meet performance specifications.

It is important to note that the present analysis is based on several simplifying assumptions and have some limitations. Firstly, the model assumes an idealized, lossless system and does not account for damping mechanisms such as material viscoelasticity or fluid viscous losses, which may significantly affect wave propagation and energy attenuation. Secondly, the formulation relies on linear assumptions for both the fluid and structural dynamics, thereby excluding nonlinear effects that may become relevant at higher excitation levels or in complex fluid-structure interactions. Finally, the study is confined to a two-dimensional axisymmetric configuration, which, while effective for symmetric structures, does not capture fully three-dimensional or asymmetric phenomena often encountered in real-world applications. Future work should aim to incorporate damping, nonlinearities, and three-dimensional geometries to enhance the model’s fidelity and applicability.

## 6 Conclusion

This article presents a detailed examination of acoustic scattering caused by a circular membrane disc embedded within a cylindrical duct, emphasizing how variations in edge boundary conditions and geometric configurations influence wave propagation. To model the interaction accurately, two complementary analytical methods were employed: the Mode Matching (MM) approach for treating rigid, non-vibrating transitions, and a Galerkin-based formulation tailored for the elastic membrane, which accommodates structural deformation and dynamic coupling with the surrounding fluid. The Galerkin strategy facilitated the use of basis functions specifically adapted to the membrane’s edge constraints, enabling an accurate representation of its vibrational response. Notably, the semi-analytical framework ensures continuity of acoustic pressure and axial velocity at the fluid–structure interfaces by employing truncated modal expansions. Although infinite series are truncated for computational practicality, the results display strong convergence behavior and preserve physical fidelity. The matching conditions at the interfaces are thereby satisfied with high precision, supporting the conservation of acoustic energy and validating the model’s reliability.

The findings reveal that edge constraint configurations significantly influence scattering behavior. When both membrane edges are subjected to symmetric spring-like restraints, the resulting acoustic profiles exhibit smooth transitions and reduced reflection, indicative of effective impedance matching and minimal modal excitation. In contrast, introducing asymmetry—especially through a free boundary—amplifies structural resonances and leads to pronounced scattering peaks due to stronger modal interactions, particularly near resonance frequencies. These behaviors were consistently observed across both frequency spectra and radial variations, confirming the robustness of the parametric trends. Additionally, modifying the membrane’s outer radius *d* was found to play a pivotal role in shaping the scattering response. Larger discs support more vibrational modes, leading to complex modal interactions and richer spectral content in the scattered field. This sensitivity to geometric and mechanical parameters suggests that careful tuning of membrane properties and boundary conditions can offer precise control over sound manipulation in duct-based systems. The study highlights the critical interplay between structural configuration and analytical formulation in accurately modeling and controlling acoustic scattering across flexible discontinuities. The proposed methodology and insights contribute to the design of reconfigurable acoustic devices such as adaptive liners, waveguide filters, and sound attenuation systems.

For future research, we recommend focused investigations into nonlinear membrane dynamics to capture large-amplitude effects, systematic incorporation of material damping models to reflect realistic energy dissipation, and exploration of multilayer membrane configurations to achieve broadband acoustic control. Furthermore, the integration of actively tunable boundary conditions through piezoelectric or smart materials could enable dynamic modulation of acoustic properties, providing pathways for next-generation adaptive waveguide technologies.
